# Clinical Characteristics and Predictors of Primary Non-Response to Biologic Therapy in Rheumatoid Arthritis and Axial Spondyloarthritis

**DOI:** 10.31138/mjr.061225.brn

**Published:** 2026-06-16

**Authors:** Kübra Sinem Beke, Hüseyin Kaplan, Gizem Cengiz, İsa Cüce, Hasan Kara, Çalış Mustafa, Mehmet Kırnap

**Affiliations:** 1 Department of Physical Medicine and Rehabilitation, Division of Rheumatology, Faculty of Medicine, Erciyes University, Kayseri, Türkiye;; 2 Department of Rheumatology, Aksaray Training and Research Hospital, Aksaray, Türkiye

**Keywords:** rheumatoid arthritis, spondylarthropathies, antirheumatic agents, treatment outcome

## Abstract

**Aim:**

According to EULAR treat-to-target recommendations, biologic (bDMARD) and targeted synthetic (tsDMARD) therapies are recommended for patients with rheumatoid arthritis (RA) and axial spondyloarthritis (axSpA) who have an inadequate response to conventional treatment. However, some patients either fail to achieve the desired response (primary non-response) or lose efficacy over time (secondary non-response). This study aimed to determine the rates and predictors of primary non-response to bDMARD/tsDMARDs in RA and axSpA.

**Methods:**

This was a retrospective study including RA and axSpA patients who received bDMARD/tsDMARD therapy at X University between January 2017 and March 2025. Primary non-response was defined as no clinical improvement within six months of treatment initiation, and secondary non-response as loss of initial response within 6–12 months (early) or after 12 months (late). Statistical analyses were performed using SPSS 23.0.

**Results:**

A total of 495 patients (167 RA, 328 axSpA) were analysed. Primary non-response was higher in RA (45.1%) than axSpA (15.9%), as was secondary non-response (80.4% vs. 39.3%, p<0.001). In RA, longer disease duration was independently associated with primary non-response (OR 1.054, 95% CI 1.008– 1.102; p = 0.021). In axSpA patients, higher BMI was independently associated with primary non-response (OR 1.079; 95% CI 1.025–1.136; p = 0.004). No other demographic or clinical variables remained significantly associated with primary non-response after multivariable adjustment in either group.

**Conclusion:**

Longer disease duration in RA and higher BMI in axSpA were independent predictors of primary non-response, highlighting the importance of early treatment in RA and metabolic considerations in axSpA.

## INTRODUCTION

Recent years have seen several significant breakthroughs in the treatment of inflammatory rheumatic diseases, with biological disease-modifying antirheumatic drugs (bDMARDs) and targeted synthetic disease-modifying antirheumatic drugs (tsDMARDs) being amongst the most notable. bDMARD/tsDMARDs have been particularly effective in the management of axial spondyloarthritis (axSpA) and rheumatoid arthritis (RA).^1,2^ These agents have been shown to suppress disease activity and reduce radiographic progression, defined as the progression of structural joint damage assessed by validated scoring systems such as ero- sion and joint space narrowing scores in RA and the modified Stoke Ankylosing Spondylitis Spine Score (mSASSS) in axSpA, thereby improving functional capacity and quality of life.^3,4^ The aforementioned effects have resulted in the integration of bDMARDs and tsDMARDs into international treatment algorithms, particularly within the European Alliance of Associations for Rheumatology (EULAR) treat-to-target recommendations for RA and the Assessment of SpondyloArthritis International Society (ASAS)-EULAR recommendations for axSpA.^5,6^ However, despite their proven efficacy, the real-world effectiveness of currently available bDMARDs and tsDMARDs may be limited in clini- cal practice due to patient non-adherence and/or early treatment discontinuation. This, in turn, results in the long-term clinical management of RA being rendered more arduous, engenders elevated costs for healthcare systems, and necessitates the development of sequential treatment strategies predicated on scant clinical and economic evidence. ^7^

The efficacy of bDMARD/tsDMARD treatment is not uniform among patients. In clinical practice, some patients fail to achieve predefined response criteria within the first six months of treatment initiation; this condition is defined as primary non-response. In contrast, secondary non-response refers to the loss of an initially achieved clinical response after this early treatment period.^8^ As primary and secondary non-responsiveness may be related to different clinical, pathophysiological processes, treatment strategies differ accordingly. In cases of primary non-response, switching to another bDMARD or tsDMARD, preferably with a different mechanism of action, is generally recommended. Conversely, in cases of secondary non-responsiveness, factors such as immunogenicity, drug levels, or patient compliance are taken into account.^9^

It is therefore vital to determine which demographic, clinical, and laboratory factors may be associated with primary non-response, so that more effective and individualised treatment strategies can be developed for the management of the disease. The present study was conducted to determine the rates of primary and secondary non-response to bDMARD/tsDMARD treatment in patients diagnosed with axSpA and RA, and to reveal the parameters associated with primary non-response. Understanding the predictors of primary non-response is clinically important for identifying high-risk patients and may also inform therapeutic algorithms in rheumatology by guiding early agent selection and treatment sequencing decisions.

## MATERIALS AND METHODS

This retrospective observational cohort study was conducted at the Outpatient Clinic of the Department of Rheumatology and the Department of Physical Medicine and Rehabilitation, Faculty of Medicine, Erciyes University, between January 1, 2017, and March 1, 2025. The study population comprised patients aged 18 years and over who had been diagnosed with RA according to the 2010 American College of Rheumatology (ACR)/EULAR classification criteria or with axSpA according to the ASAS classification criteria, and who were receiving bDMARD or tsDMARD therapy.^10,11^ Patients fulfilling these classification criteria and having available baseline and follow-up data for treatment response assessment were included in the study, with no gender discrimination. Patients with a treatment duration of less than three months, patients with incomplete data records, and patients who were lost to follow-up were excluded from the study. The study data set was created by meticulously reviewing the admission dates, clinical evaluation notes, laboratory results, and treatment protocols used through electronic patient records. All clinical and laboratory data were recorded based on the patients last rheumatology outpatient visit. The demographic data of each patient, including age, gender, height, weight, and body mass index (BMI), was meticulously recorded. Rheumatological diagnoses and comorbid diseases were also documented for each patient. The response to treatment was evaluated based on the European Alliance of Associations for Rheumatology (EULAR) response criteria and minimal disease activity (MDA) criteria. For RA patients, the EULAR response criteria based on DAS28-CRP were applied; a decrease of <0.6 or a final DAS28-CRP>5.1 after 6 months was considered non-response. For axSpA patients, response was assessed according to the Minimal Disease Activity (MDA) framework, using ASDAS-CRP scores. An ASDAS improvement <1.1 or a persistent ASDAS ≥2.1 at 6 months was defined as non-response, consistent with ASAS–EULAR recommendations. In this context, the term ‘primary non-response’ was defined as the absence of clinical response in the first six months of bDMARD/tsDMARD treatment. Patients who met these thresholds for non-response within the first six months were classified as primary non-responders, whereas those who initially met response criteria but later lost this response were categorised as secondary non-responders. Early secondary non-response was defined as the disappearance of this response within 12 months despite a response within the first 6 months of treatment. Late secondary non-response was defined as the loss of clinical efficacy after a response to treatment for more than 12 months.^8^ In the present study, patients who developed primary non-response to at least one bDMARD or tsDMARD agent were included in the ‘primary non-response’ group, and patients who initially responded to treatment with at least one agent and then lost response were included in the ‘secondary non-response’ group. The definition of primary and secondary non-response was established separately within the disease course. As independent analyses were not planned for both types of non-response, and predictor models were not created, the inclusion of the same patient in both groups did not affect the analysis design. Secondary non-response was reported exclusively in terms of frequency rate. The study size was determined by the number of eligible patients treated at our centre during the study period; no formal sample size calculation was performed.

The study protocol was approved by the Erciyes University Faculty of Medicine Hospital Ethics Committee (approval no 2025/294, dated June 4, 2025) and was carried out in accordance with the principles of the Declaration of Helsinki.

To minimise information bias, all diagnoses and treatment responses were confirmed using standardised EULAR and ASAS–EULAR criteria, and data were extracted from electronic medical records using predefined forms. Selection bias was reduced by including all consecutive eligible patients within the study period.

### Statistical Analysis

Statistical analyses were performed using IBM SPSS Statistics version 23.0 (IBM Corp., Armonk, NY, USA). Analyses were conducted separately for RA and ax- SpA patients. Descriptive statistics were presented as mean ± standard deviation or median (interquartile range) for continuous variables, depending on distribution normality assessed by visual inspection and the Kolmogorov–Smirnov test, and frequency (percentage) for categorical variables. Baseline characteristics were compared between patients with and without primary non-response using the Student’s t-test or Mann–Whitney U test for continuous variables, as appropriate, and the chi-square test for categorical variables. Univariate logistic regression analyses were performed to assess the association between baseline variables and primary non-response. Multivariable logistic regression models were subsequently constructed to identify independent predictors. Odds ratios (ORs) with 95% confidence intervals (CIs) were calculated. A two-tailed p value < 0.05 was considered statistically significant.

## RESULTS

The study comprised 167 patients with RA and 328 patients with axSpA. The median age of the RA group was 58 years (44-64), while the median age of the axSpA group was 44.7 ± 11 years (p<0.001). The proportion of women was 84.4% in the RA group and 55.2% in the axSpA group (p<0.001). Furthermore, 74.3% of RA patients exhibited comorbidity, while this proportion was reduced to 42.7% in the axSpA group (p<0.001). The most prevalent comorbid conditions were hypertension, osteoporosis, diabetes mellitus, pulmonary diseases and hyperlipidaemia, as evaluated in the patients included in the study. Hypertension (47.2%), osteoporosis (47.2%), diabetes mellitus (22.9%) and hyperlipidaemia (18.1%) were reported more frequently in RA patients (data not shown).

A comparison of first-line biologic treatment preferences between RA and axSpA patients revealed a statistically significant difference (p<0.001). Among patients with RA, 67.6% initiated treatment with a TNF inhibitor, while 32.3% started with a non-TNF biologic agent. In contrast, 97% of patients with axSpA received a TNF inhibitor as their initial biologic therapy. The primary non-response rate was significantly higher in RA patients than in axSpA patients (45.1% vs. 15.9%, p < 0.001). Similarly, late secondary non-response was more frequent in RA patients compared to axSpA patients (79.4% vs. 31.7%, p < 0.001). Conversely, no statistically significant difference was observed between the two groups concerning early secondary non-response rates (RA: 10.8%, AS: 11.3%; p=0.889). Furthermore, the utilisation of three or more bDMARD/tsDMARD was observed to be 36.3% in RA patients and 19.5% in AS patients, exhibiting a significant difference (p=0.002). The demographic and clinical characteristics of patients with RA and axSpA are presented in **Table 1**.

**Table 1.** Comparison of demographic and clinical characteristics of patients with RA and axSpA.

	**RA N=167**	**axSpA N=328**	**p**

Age	58 (44–64)	44.70 ± 11	<0.001 ^*^

Female, n (%)	141 (84.4)	181 (55.2)	<0.001 ^*^

BMI (kg/m^2^)	30.03 ± 6.41	28.22 (25.12–32)	0.210

Disease Duration (years)	15 (10–20)	8 (3–12)	<0.001 ^*^

Smoking Status, yes n (%)	28 (16.8)	88 (26.8)	0.012^*^

Comorbidity, yes n (%)	124 (74.3)	140 (42.7)	<0.001 ^*^

RF, positive, yes n (%)	116 (69.5)		

RF Titre	43.2 (8.8–121.7)		

Anti CCP, positive, yes n (%)	115 (68.9)		

Anti-CCP Titre	214 (8–500)		

HLAB27, positive, yes n (%)		145 (44.2)	

DAS28	2.5 (2–3.8)		
ASDAS-CRP		1.6 (1.3–2.2)	

ASDAS-ESR		2 (1.6 – 2.5)	

BASDAI		2.1 (2–3)	

ESR (mm/hour)	21 (11–33)	11 (6–26)	<0.001 ^*^

CRP (mg/L)	4.66 (1.33–10.55)	2.44 (1.11–6.58)	0.006^*^

INH Prophylaxis, yes n (%)	83 (49.7)	231 (70.4)	<0.001 ^*^

Hepatitis Prophylaxis, yes n (%)	44 (26.3)	40 (12.2)	<0.001 ^*^

Current bDMARD			

Anti-TNF	87(52)	303 (92.3)	

IL-6 Inhibitor	24 (14.4)		

CTLA-4 Inhibitor	13 (7.8)		

Anti-CD20	11 (6.6)		

JAK Inhibitor	32 (19.1)	1 (0.3)	

IL-17 Inhibitor		24 (7)	

Total Duration of Biologic Use, years	8.75 ( 5.25	4.25 (1.5–8 )	<0.001 ^*^

First Preferred Biologic Agent			
TNF Inhibitor	113 (67.6)	320 (97)	0.001^*^
Non-TNF Biologic	54 (32.3)	8 (3)	

First Biologic Drug Survival Duration	1.75 (0.5–5)	2 (0.75–5)	0.328

Biologic Preferred After First Agent			
TNF – TNF^*^	41 (40.2)	145 (87.9)	<0.001 ^*^
TNF Non-TNF	31 (30.4)	17 (10.3)	
Non-TNF- TNF	17 (16.7)	1 (0.6)	
Non-TNF- Non-TNF	13 (12.7)	2 (1.2)	

Primary Non-Response, yes n (%)	46 (45.1)	52 (15.9)	<0.001 ^*^

Secondary Non-Response, yes n (%)	82 (80.4)	129 (39.3)	<0.001 ^*^

Early Secondary Non-Response, yes n (%)	11 (10.8)	37 (11.3)	0.889

Late Secondary Non-Response, yes n (%)	81 (79.4)	104 (31.7)	<0.001 ^*^

Reasons for Drug Discontinuation			0.401
Lack of Efficacy	212	253	
Adverse Event	20	25	
Other	10	6	

Number of bDMARDs Used			0.002^*^
1	39 (38.2)	163 (49.7)	
2	26 (25.5)	101 (30.8)	
3 or More	37 (36.3)	64 (19.5)	

n: number of patients, RA: Rheumatoid Arthritis; axSpA: Axial Spondyloarthritis; RF: Rheumatoid Factor; CCP: Cyclic Citrullinated Peptide Antibody; HLA-B27: Human Leukocyte Antigen B27; DAS28: Disease Activity Score 28; ASDAS: Ankylosing Spondylitis Disease Activity Score; BASDAI: Bath Ankylosing Spondylitis Disease Activity Index; ESR: Erythrocyte Sedimentation Rate; CRP: C-reactive protein; INH: Isoniazid; bDMARD: Biologic Disease-Modifying Antirheumatic Drug; TNF: Tumour Necrosis Factor; JAK: Janus Kinase; CTLA-4: Cytotoxic T-Lymphocyte Antigen 4.

Demographic and clinical characteristics of RA patients with and without primary response were compared. No significant difference was found between the groups in terms of age, gender, BMI, and disease duration (p>0.05 for all variables). However, the duration of the first bDMARD/tsDMARD treatment was found to be significantly shorter in patients who developed primary non-response (median 0.5 years vs. 1.75 years; p<0.001). Furthermore, the use of three or more bDMARD/tsDMARD was found to be significantly more prevalent in these patients (p <0.001). While disease duration (p = 0.066) and total duration of bDMARD/tsDMARD use (p = 0.054) were longer in primary non-responders, these differences did not attain statistical significance. A detailed comparison of the relevant data is presented in **Table 2**.

**Table 2.** Comparison of demographic, clinical, and treatment characteristics between RA patients with and without primary non-response.

	**Patients without Primary Non-Response N= 121**	**Patients with Primary Non-Response N=46**	**p**

Age	59 (47–64)	49.73 ± 14.06	0.521

Female, n (%)	101 (83.5)	40 (87)	0.579

BMI (kg/m^2^)	29.56 ± 0.8	29.90 ± 6.17	0.747

Disease Duration (years)	13 (10–17)	15 (10–20)	0.066

Smoking Status, yes n (%)	19 (15.7)	9 (19.6)	0.551

Comorbidity, yes n (%)	90 (74.4)	34 (73.9)	0.951

RF, positive, yes n (%)	84(69.4)	32(69.6)	

RF Titre	45.30 (12.45–123.45)	31.3 (8.07–129.55)	0.584

Anti CCP, positive, yes n (%)	87 (71.9)	28 (60.9)	0.149

Anti-CCP Titre	200 (8–500)	234 (8–500)	0.583

First Biologic Drug Survival Duration	1.75 (0.87–3.62)	0.5 (0.25–1)	<0.001^*^

ESR at the Initiation of Biologic Therapy (mm/hour)	31(21.5–42.5)	28 (21–46)	0.780

CRP at the Initiation of Biologic Therapy (mg/L)	20.7 (10.23–41.33)	14.95 (8.11–25.20)	0.260

DAS28 at the Initiation of Biologic Therapy	6.15 ± 0.56	6.13 ± 0.93	0.771

Total Duration of Biologic Therapy Use, years	6.25 (2–10)	8.75 (5.5–10.25)	0.054

First Preferred Biologic Agent			0.214
TNF Inhibitor	80 (66.1)	35 (76.1)	
Non-TNF Biologic	41 (33.9)	11 (23.9)	

Number of bDMARDs Used			
1	27 (48.2)	12 (26.1)	
2	21 (37.5)	5 (10.9)	<0.001^*^
3 or More	8 (14.3)	29 (63)	

n: number of patients, RA: Rheumatoid Arthritis; RF: Rheumatoid Factor; CCP: Cyclic Citrullinated Peptide Antibody; ESR: Erythrocyte Sedimentation Rate; CRP: C-reactive protein; DAS28: Disease Activity Score in 28 joints; bDMARD: Biologic Disease-Modifying Antirheumatic Drug; TNF: Tumour Necrosis Factor.

Univariate logistic regression analysis was performed to evaluate baseline factors associated with primary non-response in patients with RA. Among the examined variables, disease duration was significantly associated with primary non-response (OR: 1.047; 95% CI: 1.003–1.093; p = 0.037). Age, gender, BMI, smoking status, seropositivity, and baseline DAS28 at the initiation of biologic therapy were not significantly associated with primary non-response (all p > 0.05).

In multivariable logistic regression analysis adjusted for age, disease duration remained independently associated with primary non-response (OR: 1.054; 95% CI: 1.008–1.102; p = 0.021), whereas age was not significantly associated with the outcome (p = 0.176) (**Table 3**).

**Table 3.** Univariate and multivariate logistic regression analysis of variables associated with the development of primary non-response in patients with RA.

	**Primary Non-Response in RA**			
	**Univariate**		**Multivariate**	
	**OR (95% CI)**	**p**	**OR (95% CI)**	**p**
Age (years)	0.987 (0.960–1.016)	0.380	0.980 (0.951–1.009)	0.176
Gender (Female)	1.320 (0.494–3.528)	0.580		
BMI (kg/m^2^)	1.009 (0.955–1.067)	0.745		
Smoking Status, yes	0.766 (0.318–1.842)	0.551		
Disease Duration (years)	1.047 (1.003–1.093)	0.037^*^	1.054 (1.008–1.102)	0.021^*^
Seropositivity, yes	1.050 (0.500–2.204)	0.897		
DAS28 at the Initiation of Biologic	0.930 (0.570–1.517)	0.770		

n: number of patients, RA: Rheumatoid Arthritis; DAS28: Disease Activity Score in 28 joints.

A comparison was made of the demographic and clinical characteristics of axSpA patients with and without primary response, but no significant difference was found between the groups in terms of age, gender and disease duration (p>0.05 for all variables). However, a significant difference was observed in the ASDAS-CRP (p = 0.004) and ASDAS-ESR (p <0.001) scores between patients with axSpA who did and did not develop primary non-response. Furthermore, analysis indicated a significant increase in BMI among patients who exhibited primary non-response (p = 0.003). The present study found that human leucocyte antigen B27 (HLA-B27) positivity was significantly higher in patients who did not develop a primary response (46.7% vs. 30.8%; p = 0.023). Furthermore, the duration of initial bDMARD/tsDMARD treatment was found to be significantly shorter in patients who did not respond to treatment (median 0.25 years vs. 1.75 years; p <0.001). Detailed data on other comparisons can be found in **Table 4**.

**Table 4.** Comparison of demographic, clinical, and treatment characteristics between axSpA patients with and without primary non-response.

	**Patients without Primary Non-Response N= 276**	**Patients with Primary Non-Response N=52**	**p**

Age	44.66 ± 11.19	44.88 ± 10.47	0.893

Female. n (%)	146 (52.9)	35 (67.3)	0.055

BMI (kg/m^2^)	27.82 (24.96–31.47)	30.10 (27.39–35.45)	0.003^*^

Disease Duration (years)	8 (3–12)	5.5 (2.25–10)	0.103

Smoking Status. yes n (%)	77 (27.9)	11 (21.2)	0.314

Comorbidity. yes n (%)	112 (40.6)	28 (53.8)	0.076

HLAB27. positive. yes n (%)	129 (46.7)	16 (30.8)	0.023^*^

First biologic Drug Survival Duration	1.75 (1–3.06)	0.25 (0.25–0.75)	<0.001^*^

ESR at the Initiation of Biologic Therapy (mm/hour)	18.5 (8–33)	23 (6–34)	0.875

CRP at the Initiation of Biologic Therapy (mg/L)	9.71 (2.63–20.87)	5.89 (1.65–16.1)	0.124

BASDAI at the Initiation of Biologic Therapy	6.7 (6.17–7.22)	6.80 (6.10–7.5)	0.941

ASDAS CRP	1.6 (1.3–2)	1.9 (1.5–2.5)	0.004^*^

ASDAS ESR	2 (1.6–2.5)	2.5 (2–3)	<0.001^*^

Total Duration of Biologic Therapy Use, years	4.62 (1.75–8)	3.37 (1.25–8.5)	<0.001^*^

First Preferred Biologic Agent			0.909
TNF Inhibitor	270 (97.8)	51 (98.1)	
Non-TNF Biologic	6 (2.2)	1 (1.9)	

Number of bDMARDs Used			<0.001^*^
1	163 (59.1)	0 (0)	
2	85(30.8)	16 (30.8)	
3 or More	28 (10.1)	36 (69.2)	

n: number of patients; axSpA: Axial Spondyloarthritis; HLA-B27: Human Leukocyte Antigen B27; ESR: Erythrocyte Sedimentation Rate; CRP: C-reactive protein; BASDAI: Bath Ankylosing Spondylitis Disease Activity Index; ASDAS: Ankylosing Spondylitis Disease Activity Score; bDMARD: Biologic Disease-Modifying Antirheumatic Drug; TNF: Tumour Necrosis Factor.

In the univariate logistic regression analysis performed to identify baseline factors associated with primary non-response in patients with axSpA, higher BMI (OR: 1.079; 95% CI: 1.030–1.171; p = 0.001), higher baseline ASDAS-CRP scores (OR: 1.82; 95% CI: 1.145–2.896; p = 0.011), and HLA-B27 positivity (OR: 0.426; 95% CI: 0.220–0.824; p = 0.011) were significantly associated with primary non-response. Female gender showed a borderline association (OR: 1.833; 95% CI: 0.980–3.427; p = 0.058), whereas age, smoking status, and disease duration were not significantly associated with the outcome (all p> 0.05).

In the multivariable logistic regression analysis, only higher BMI remained independently associated with primary non-response (OR: 1.079; 95% CI: 1.025–1.136; p = 0.004). HLA-B27 positivity did not retain statistical significance in the multivariate model (OR: 0.531; 95% CI: 0.263–1.071; p = 0.077), and neither gender (p = 0.628) nor baseline ASDAS-CRP (p = 0.704) were independently associated with primary non-response (**Table 5**).

**Table 5.** Univariate and multivariate logistic regression analysis of variables associated with the development of primary non-response in patients with axSpA.

	**Primary Non-Response in axSpA**			
	**Univariate**		**Multivariate**	
	**OR (95% CI)**	**p**	**OR (95% CI)**	**p**
Age (years)	1.002 (0.975–1.029)	0.893		
Gender (Female)	1.833 (0.980–3.427)	0.058	0.832 (0.396–1.750)	0.628
BMI (kg/m^2^)	1.079 (1.030–1.171)	0.001^*^	1.079 (1.025–1.136)	0.004^*^
Smoking Status, yes	1.442 (0.705–2.950)	0.316		
Disease Duration (years)	0.969 (0.919–1.021)	0.236		
ASDAS-CRP at the Initiation of Biologic	1.82 (1.145–2.896)	0.011^*^	1.068 (0.760–1.501)	0.704
HLAB27 positive. yes	0.426 (0.220–0.824)	0.011^*^	0.531 (0.263–1.071)	0.077

n: number of patients; axSpA: Axial Spondyloarthritis; ASDAS: Ankylosing Spondylitis Disease Activity Score; CRP: C-reactive protein; HLA-B27: Human Leukocyte Antigen B27.

## DISCUSSION

In this large real-world cohort, primary non-response to bDMARD/tsDMARD therapy was observed in 45.1% of patients with RA and 15.9% of patients with axSpA, indicating a markedly higher early treatment failure rate in RA. In addition, late secondary non-response was substantially more frequent in RA compared with ax-SpA (79.4% vs. 31.7%), further highlighting differences in long-term treatment sustainability between the two diseases. These findings suggest that, despite similar therapeutic algorithms, the real-world effectiveness of biologic and targeted synthetic agents may differ considerably between RA and axSpA populations. Importantly, the predictors of primary non-response also differed between the two conditions. In RA, longer disease duration was independently associated with primary non-response, supporting the concept that prolonged inflammatory burden and cumulative structural damage may reduce treatment responsiveness. In contrast, in axSpA, higher BMI was the only independent predictor after multivariable adjustment, emphasising the potential impact of metabolic factors on biologic efficacy. Although baseline ASDAS-CRP and HLA-B27 status showed associations in univariate analyses, these did not persist in the adjusted model, suggesting that metabolic status may play a more dominant role than classical disease activity markers in determining early treatment failure in axSpA.

Although rheumatoid arthritis is generally more prevalent than axial spondyloarthritis in the general population, our cohort included a higher number of axSpA patients. This discrepancy may be explained by the inclusion of only patients receiving bDMARD/tsDMARD therapy. In routine clinical practice, a substantial proportion of RA patients may achieve adequate disease control with conventional synthetic DMARDs and therefore are not escalated to biologic therapy. In contrast, axSpA patients more frequently require biologic treatment according to current treatment algorithms, which may have contributed to the higher number of axSpA patients in our study population.

In the present study, it was observed that patients suffering from RA were older and predominantly female when compared to patients suffering from axSpA. This finding was consistent with the literature.^12,13^ It was also observed that patients suffering from RA had a higher burden of comorbidities. While 74.3% of RA patients had at least one comorbidity, this rate was 42.7% in axSpA patients. This discrepancy can be attributed, at least in part, to the fact that age-related comorbidities are more prevalent in RA, given that the onset of RA occurs at an older age and is more frequently observed in women. Furthermore, the chronic nature of RA, characterised by systemic inflammation, has been demonstrated to facilitate the development of comorbidities, including cardiovascular, metabolic and psychiatric diseases.^14,15^ In the present study, cardiovascular and metabolic diseases were identified as the most prevalent comorbidities in the RA group. The present findings serve to underscore the necessity of meticulous monitoring of cardiovascular risks and metabolic comorbidities in patients suffering from RA.

A comparison of biologic treatment preferences between RA and axSpA patients revealed that, among those with RA, the first-line biologic agent was a TNF inhibitor in 113 patients (67.6%), while 54 patients (32.3%) were initiated on a non-TNF agent. These findings suggest that although TNF inhibitors remain the most frequently preferred initial therapy in RA, nearly one-third of patients received a non-TNF biologic, likely reflecting individualised treatment decisions based on clinical and serological characteristics. In contrast, TNF inhibitors were overwhelmingly preferred as the initial biologic treatment in axSpA patients (97%). These observations can be attributed to the divergent pathogenesis and treatment algorithms characteristic of these two diseases.^5,6^ Furthermore, it has been observed that a wider range of biological agents is utilised in RA patients, whereas treatment in axSpA patients is largely confined to TNF inhibitors. This discrepancy can be attributed to the availability of drug options with different mechanisms of action for RA, the diversity of patient preferences, and the presence of comorbidities, which may influence the choice of treatment.

The duration of initial biological agent use was similar between the two groups (RA: 1.75 years, axSpA: 2 years), but differences in treatment transitions were noteworthy. When a change of medication is necessary due to ineffectiveness, side effects, or other reasons after the first bDMARD/tsDMARD treatment, there is still insufficient evidence regarding which medication should be preferred. In this process, physicians generally take treatment guidelines and current efficacy data into account.^16^ Despite the existence of studies indicating that transitioning from one anti-TNF agent to another can yield favourable outcomes, some data that calls into question the efficacy of this approach.^17,18^ Two well-known fundamental mechanisms of treatment failure that develop against initial TNF inhibitors are known as primary and secondary non-response. Secondary non-response is often associated with pharmacokinetic issues, such as immunogenicity and reduced drug levels, whereas primary non-response may indicate inadequate targeting of the dominant inflammatory pathway. In addition to these mechanisms, patient adherence may also influence treatment outcomes, particularly in real-world settings, where irregular drug use or premature discontinuation may contribute to apparent treatment failure.^19^

In the present study, transitions between TNF and non-TNF agents in the RA group occurred more frequently and in a more varied manner. However, treatment transitions in the axSpA group predominantly took place between TNF inhibitors.

Upon examination of primary and secondary non-response rates, it was observed that in both disease groups, the secondary non-response rates were higher than the primary non-response rates. These observations were consistent with the extant literature.^20, 21^ In patients diagnosed with RA, the incidence of primary non-response (45.1%) and secondary non-response (80.4%) was higher in comparison to patients diagnosed with axSpA (15.9% and 39.3%, respectively). Furthermore, late secondary non-response was observed more frequently in both groups compared to early secondary non-response; particularly in RA patients, the rate of late response loss (79.4%) was significantly higher than in the axSpA group (31.7%). The findings of this study underscore the significance of commencing treatment with an appropriate agent at an early stage, a principle that is especially salient in the context of RA patients, whilst concomitantly taking into account patient-specific risk factors.

In addition, the use of three or more bDMARD/tsDMARDs was significantly higher in the RA group than in the axSpA group (36.3% vs. 19.5%), indicating that more treatment switches were required in RA patients. According to the EULAR treat-to-target recommendations, treatment response should be assessed regularly, and therapeutic adjustments are recommended if predefined targets are not achieved within an appropriate time frame.^5,6^ These recommendations highlight the importance of early evaluation of treatment effectiveness and timely modification of therapy in patients who fail to respond adequately.

In RA patients, longer disease duration emerged as an independent factor associated with primary non-response. This finding may reflect the cumulative impact of sustained inflammatory activity, progressive structural damage, and prior therapeutic exposure over time. Several studies have suggested that delayed initiation of effective biologic therapy is associated with reduced probability of achieving optimal clinical response, supporting the concept of a “window of opportunity” in RA management.^22^ Consistent with the EULAR treat-to-target recommendations, early and sustained suppression of disease activity is associated with improved long-term outcomes.^5^ Therefore, our results reinforce the importance of timely treatment optimisation, particularly in patients with long-standing RA.

A significant association between obesity and autoimmune diseases has been documented in the literature.^23^ As demonstrated in several investigations, there is an elevated risk of developing inflammatory diseases such as axSpA, psoriasis, and psoriatic arthritis in obese individuals.^24, 25^ The underlying mechanisms that precipitate this relationship are attributable to the heightened pro-inflammatory cytokines that are secreted from adipose tissue, in conjunction with the augmented expression of adipokines.^26^ In the present study, no significant association was observed between BMI and primary non-response in patients with RA. In contrast, findings from the axSpA cohort were consistent with previous literature. Patients with axSpA who developed primary non-response had significantly higher BMI values, and both univariate and multivariable logistic regression analyses confirmed elevated BMI as an independent predictor of primary non-response. These results suggest that obesity may not only contribute to disease pathogenesis but also adversely influence treatment responsiveness, potentially through mechanisms involving altered pharmacokinetics, chronic low-grade inflammation, and cytokine dysregulation.

However, this study has several limitations. Due to its retrospective design, some clinically important data, such as treatment adherence, could not be obtained and incorporated into the analysis. Non-adherence might therefore represent a potential confounding factor that could have led to the misclassification of some cases as primary non-response. In addition, as this was a single-centre study, the generalisability of the findings may be limited. The very high proportion of anti-TNF-α use as first-line biologic therapy in axSpA (97%) reflects local prescribing patterns and may have introduced treatment selection bias. The absence of data on drug levels and immunogenicity complicates the detailed elucidation of the biological mechanisms underlying primary non-response. Nevertheless, the absence of standard and complete data records regarding the durations between the commencement of treatment and the onset of non-responsiveness precludes the application of time-based analysis methods (e.g. Kaplan-Meier, Cox regression). Consequently, primary non-response was evaluated as a binary (yes/no) outcome variable in the study.

Despite these limitations, the present study also has several important strengths. First, it includes a relatively large, well-characterised real-world cohort of both RA and axSpA patients treated in routine clinical practice, allowing a direct comparison of patterns and predictors of primary non-response across two major inflammatory rheumatic diseases. Second, treatment response and disease activity were assessed using standardised EULAR and ASAS–EULAR criteria and routinely used composite indices, and we systematically differentiated between primary and early/late secondary non-response, providing a more nuanced and clinically relevant picture of biologic treatment failure than most previous observational studies.

In conclusion**,** primary non-response was more frequent in RA, and treatment switches were both more common and more heterogeneous in these patients. In both diseases, secondary non-response—particularly late secondary non-response—was observed more frequently than primary non-response. In RA patients, longer disease duration independently increased the risk of primary non-response, whereas in axSpA patients, elevated BMI emerged as an independent risk factor. These findings underscore the importance of early therapeutic intervention in RA and the consideration of metabolic factors such as body mass in axSpA. Careful patient stratification and closer monitoring at treatment initiation may improve treatment outcomes. Future prospective studies incorporating pharmacokinetic data and immunogenicity markers are warranted to further clarify the mechanisms underlying primary non-response.

## Data Availability

The datasets used and/or analysed during the current study are available from the corresponding author upon reasonable request.
